# Cryotolerance strategies of Pseudomonads isolated from the rhizosphere of Himalayan plants

**DOI:** 10.1186/2193-1801-2-667

**Published:** 2013-12-12

**Authors:** Shekhar Chandra Bisht, Gopal Kishna Joshi, Shafiul Haque, Pankaj Kumar Mishra

**Affiliations:** Department of Biotechnology, HNB Garhwal University (A Central University), Srinagar, 246174 Uttarakhand India; Microbiology & Chemistry Lab, Vivekananda Institute of Hill Agriculture, (ICAR), Almora, 263601 Uttarakhand India; Department of Biosciences, Jamia Millia Islamia (A Central University), New Delhi, 110025 India

**Keywords:** Psychrotrophic, *Pseudomonas*, Cold tolerance, Raffinose, Exopolysaccharide, Free amino acids

## Abstract

The cold stress biology of psychrotrophic *Pseudomonas* strains isolated from the rhizosphere of Himalayan plants have been explored to evaluate their cryotolerance characteristcs. *Pseudomonas* strains were examined for stress metabolites, viz., exopolysaccharide (EPS) production, intracellular sugar, polyols and amino acid content, ice nucleation activity, and their freezing survival at -10 and -40°C, respectively. High freezing survival was observed for the *Pseudomonas* strains that were grown at 4°C prior to their freezing at -10 or -40°C. Increased EPS production was noticed when *Pseudomonas* strains were grown at lower temperatures, i.e., 4 and 15°C, in comparison with their optimal growth temperature of 28°C. All *Pseudomonas* strains showed low level of type-III class ice nucleation activity at -10°C after 96 h. Considerable differences were noticed in accumulated contents of various intracellular sugars, polyols, amino acids for all *Pseudomonas* strains when they grown at two different temperatures, i.e., 4 and 28°C, respectively. The unusual complement of stress protectants especially, raffinose, cysteine and aspartic acid that accumulated in the bacterial cells at low temperature was novel and intriguing finding of this study. The finding that raffinose is a key metabolite accumulated at low temperature is an exciting discovery, and to the best of our information this is first report ever signifying its role in bacterial cold tolerance.

**Electronic supplementary material:**

The online version of this article (doi:10.1186/2193-1801-2-667) contains supplementary material, which is available to authorized users.

## Background

Microorganisms have a range of evolutionary adaptations and physiological acclimation mechanisms that allow them to survive and remain active in the conditions of environmental stress. Adaptation towards stress condition is indispensable for survival, mainly when it causes alterations to the cell metabolism. Sudden decrease in temperature has severe effects on microbial cells, like, reduction of membrane fluidity, decrease in ribosome efficiency, and increased stabilization of secondary structures of nucleic acids, which may affect transcription, translation and DNA replication (Phadtare et al. 2000). In order to survive under freezing conditions, bacteria have developed various strategies for their endurance, such as, maintenance of membrane fluidity, constant metabolic activities etc. (Ramos et al. 2001). Additionally, it has been suggested that trehalose, glycerol and sorbitol are the major cryoprotectants for prokaryotic cells to response the freezing damage, thereby causing the maintenance of some enzymatic functions *in*-*vivo* (Yamashita et al. 2002). However, a limited information is available about the cryoprotectants that are responsible for the freezing resistance mechanisms of bacteria. Bacteria often encounter freezing conditions and can survive in extremely cold environments, like, the high altitude regions of Himalaya. In frozen environments, bacteria are exposed to conditions that necessitate the removal of water to maintain the structure and function of the bacterial cell. As water contributes to the stabilization of various macromolecular structures, any significant deviation from the accessibility of water due to dehydration, desiccation or an alteration of its physical state from aqueous phase to an ice crystal form poses a severe threat to the normal cell functions and survival of organism (Beall 1983; Crowe et al. 1984).

In this regard, regulatory proteins and key metabolic enzymes require adjustments to cope with the temperature shifts in order to maintain a balanced microbial growth at the new environmental temperature. Under such conditions, the synthesis of specific cryoprotectant molecules might be enhanced that act as chemical chaperons and protect the cellular proteins from freezing temperature. Scanty reports are available on psychrotrophic bacterial cryotolerance strategies and related responsible molecules except cold shock (Csps) and cold acclimation (Caps)s proteins. Although, bacterial cryotolerance has been investigated in relevance with role of trehalose and glycine betine in *Escherichia coli* and *Bacillus subtilis*, respectively (Jones et al. 1987; Willimsky et al. 1992). But, very little is known about the possibility of other molecules responsible for the survival of bacteria subjected to freezing challenge by an adaptation of the microbial cells to low temperatures particularly in psychrotolerant/psychrophilic bacteria (Margesin and Schinner 1999; Mishra et al. 2010).

In the upper parts of north west (NW) Himalaya, winter is mostly characterized by intermittent snow cover (November to March) and fluctuating subfreezing temperatures, while summer displays intense desiccating sunshine punctuated by infrequent rains (Mishra et al. 2008; 2011; Bisht et al. 2013). These conditions pose additional challenges to microbial species that may endure summer temperatures as high as 30°C and winter temperatures that can dip to -10°C, as well as alternating freezing and thawing periods during the cold season. At these temperatures, microorganisms might be injured or killed as a result of cold shock, freezing, prolonged exposure to subzero temperatures, and subsequent warming, and injury or death is often due to damage to membranes or cell walls that results in changes in permeability, as well as damage to DNA. Given these challenges, the fact that soil bacteria thrive in NW Himalayan regions is a testimony either to environmental heterogeneity or to the remarkable adaptive abilities of these psychrotrophic microbes (Srinivas et al. 2011; Bisht et al. 2013). The precise mechanisms or molecular strategies underlying the cellular adaptations of psychrotrophic bacterial cells in cold conditions are not clear and needs to be addressed, particularly for varying genus of *Pseudomonas*. In this context, the present study was undertaken to investigate the freezing survival strategies operated in six psychrotrophic *Pseudomonas* strains (*P. lurida* NPRs3, *P. lurida* NPRp15, *P.* sp. PPERs23, *P. putida* PGRs4, *P.* sp. PGERs17 and *P. fluorescens* PPRs4) isolated previously from rhizosphere of NW Himalayan plants (Mishra et al. 2011; Bisht et al. 2013).

## Results

### Bacterial growth and freeze survival

**Figure 1:**
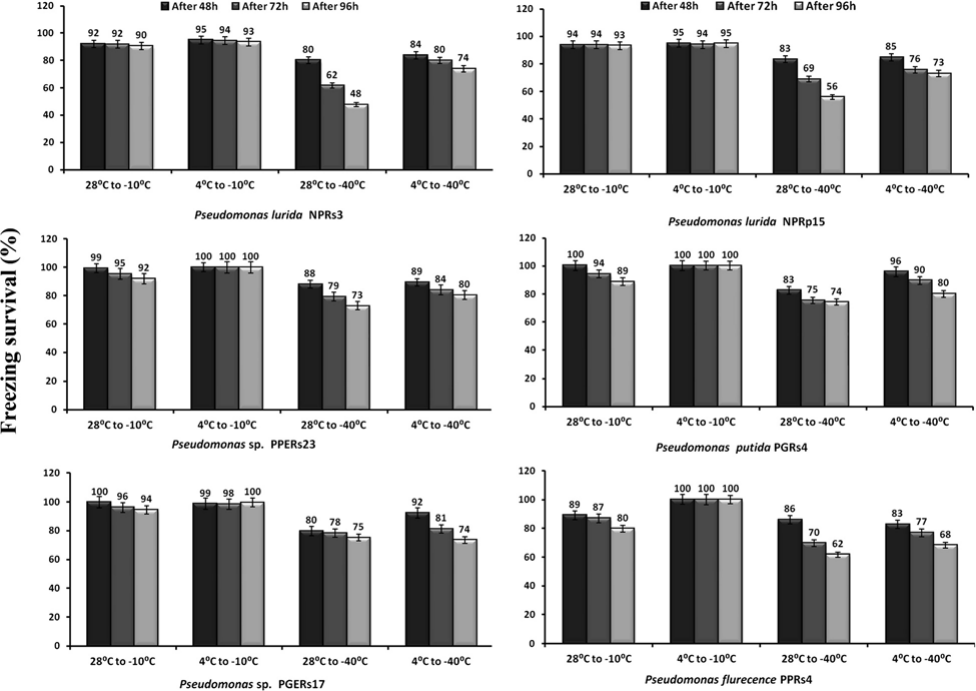
**Percentage survival of Pseudomonads subjected to freezing temperature [-10 and -40°C] shifted from two different incubation temperatures (4 and 28°C).** Note: All values are mean of three independent replicates and bar represents the standard error of mean.

Freezing survival studies of *Pseudomonas* strains revealed that strains which were grown at 4°C prior to freezing separately at -10 and -40°C demonstrated significantly higher freezing survival rather than cultures which were grown at 28°C prior to freezing (Figure 1). It was observed that *Pseudomonas* strains grown at low temperature (e.g., 4°C) have a survival advantage upon freezing tolerance compared to their optimal growth temperature (28°C).

### EPS production

**Figure 2:**
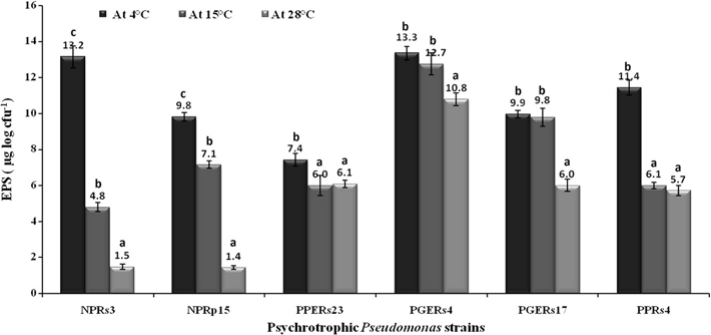
**EPS accumulation by** ***Pseudomonas*** **strains at three different incubation temperatures (4, 15 and 28°C).** Note: All values are mean of three independent replicates and bar represents the standard error of mean. The alphabet letters (a, b, c) in the column for individual *Pseudomonas* strain indicate significant differences at 4, 15 and 28°C incubation temperature.

EPS production was found to be higher at lower incubation temperatures (4 or 15°C) in comparison to the optimal growth temperature (28°C) in all the *Pseudomonas* strains (Figure 2). At 4°C, *P. lurida* NPRs3 produced 2.75 and 8.8 folds higher EPS in comparison to EPS produced at 15 and 28°C, respectively. At 15°C, *P. lurida* NPRs3 showed 3.2 folds higher EPS production in comparison with the same cells grown at 28°C. Similarly, the *P. lurida* NPRp15 cells demonstrated 1.38 and 7.0 folds higher EPS production at 4°C compared to the cells grown at 15 and 28°C, respectively. At 15°C, the *P. lurida* NPRp15 culture produced 5.07 folds greater EPS as compared to culture grown at 28°C. Likewise, the cells of *Pseudomonas* sp. PPERs23 grown at 4°C showed 23.33 and 21.31% higher EPS accumulation as compared to the cells cultivated at 15 and 28°C, respectively. However, the *Pseudomonas* sp. PPERs23 culture grown at 15 and 28°C showed almost similar EPS accumulation (Figure 2). Similarly, the *P. putida* PGRs4 cells showed 23.14% greater EPS accumulation at 4°C in comparison to the *P. putida* PGRs4 cells grown at 28°C. At 15°C, the *P. putida* PGRs4 culture showed 17.6% enhanced EPS production than the *P. putida* PGRs4 cells cultivated at 28°C. Likewise, the cells of *Pseudomonas* sp. PGERs17 cultivated at 4°C showed 66.0% increased EPS production in comparison to the cells grown at 28°C, whereas, the *Pseudomonas* sp. PGERs17 cells separately grown at 4 and 15°C failed to show significant difference in EPS accumulation. *P. fluorescens* PPRs4 demonstrated almost double amount of EPS production at 4°C growth temperature in comparison to cells grown at 15 or 28°C.

### INA of psychrotolerant *Pseudomonas* strains

The INA of Pseudomonads had been measured to determine the catalytic sites present in the bacterial cells responsible for ice formation. None of the collected strains demonstrated type-I or type-II category INA measured at -5°C. All Pseudomonads showed low level of type-III INA (active between -7 to -10°C) measured at -10°C after 24, 72 and 96 h of bacterial growth (Table 1). The type-III INA of all *Pseudomonas* strains was found to be low at long (96 h) incubation of the culture. Whereas, the same was noted high for cultures incubated for short (24 h) time period. The highest INA was found in *P. fluorescens* PPRs4 and the lowest INA was observed in *Pseudomonas* sp. PGERs17 after 24 h incubation period. The mean INA (log ice nuclei CFU^-1^) of *P. lurida* NPRs3, *P. lurida* NPRp15, and *P. fluorescens* PPRs4 was noticed to be higher at 4°C after 24 h growth incubation in comparison to 96 h of bacterial growth incubation at the same temperature. Whereas, after 24 h growth incubation at 4°C, *Pseudomonas* sp. PPERs23, *P. putida* PGRs4, and *Pseudomonas* sp. PGERs17 showed difference of 0.85, 0.97 and 0.77 log ice nuclei CFU^-1^, respectively, as compared to 96 h growth incubation at 4°C. These differences indicated lowering in INA of all *Pseudomonas* strains with long incubation time (96 h) in cold conditions (e.g., 4°C).

**Table 1:** **Ice nucleation activity of cold tolerant** ***Pseudomonas*** **strains at -10°C temperature**

Cold tolerant ***Pseudomonas*** strains	Culture incubation at 4°C														
	24 h					72 h					96 h				
	Ice nucleation activity of bacterial culture at -10°C (log ice nuclei cfu^-1^)														
	After 5 min	After 10 min	After 15 min	After 20 min	Mean	After 5 min	After 10 min	After 15 min	After 20 min	Mean	After 5 min	After 10 min	After 15 min	After 20 min	Mean
***P. lurida*** **NPRs3**	-8.47	-8.47	-8.47	-7.36	**-7.15**	-9.19	-9.19	-8.89	-8.49	**-8.11**	-9.17	-9.17	-8.8	-8.75	**-8.47**
***P. lurida*** **NPRp15**	-7.43	-7.43	-7.33	-7.24	**-7.17**	-8.49	-8.49	-8.14	-8.08	**-8.01**	-8.96	-8.96	-8.78	-8.62	**-8.56**
***Pseudomonas sp.*** **PPERs23**	-6.68	-6.68	-6.68	-6.68	**-6.38**	-7.9	-7.9	-7.9	-7.56	**-7.26**	-7.74	-7.74	-7.74	-7.43	**-7.23**
***P. putida*** **PGRs4**	-7.58	-7.40	-6.82	-6.65	**-6.18**	-7.33	-7.24	-7.17	-7.17	**-7.36**	-8.49	-8.14	-8.08	-7.15	**-7.15**
***Pseudomonas sp.*** **PGERs17**	-7.92	-7.82	-7.78	-7.62	**-7.52**	-8.47	-8.37	-8.27	-8.24	**-8.07**	-8.89	-8.59	-8.69	-8.49	**-8.29**
***P. fluorescens*** **PPRs4**	-6.82	-6.65	-5.86	-5.80	**-5.88**	-8.49	-7.92	-6.82	-6.65	**-6.65**	-8.58	-8.19	-7.9	-7.56	**-7.26**

### Accumulation of intracellular sugars and polyols

**Figure 3:**
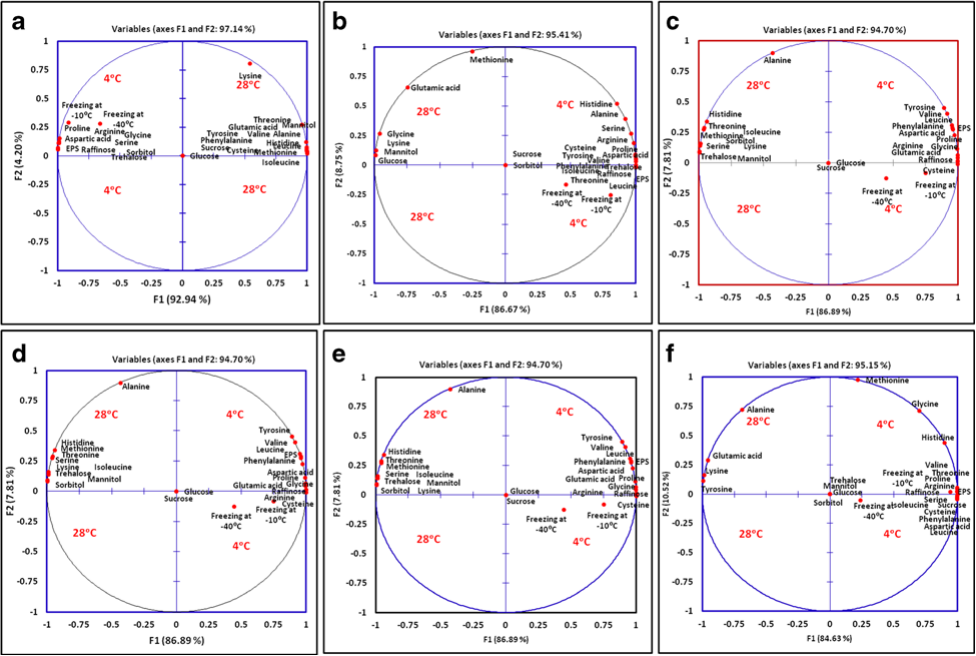
**Principal component analysis (PCA) of stress metabolites profile of** ***Pseudomonas*** **strains grown at 4 and 28°C. [a]** *P. lurida* NPRs3 **[b]** *P. lurida* NPRp15 **[c]** *Pseudomonas* sp. PPERs23 **[d]** *P. putida* PGRs4 **[e]** *Pseudomonas* sp. PGERs17 **[f]** *P. fluorescens* PPRs4. (Factor map of rows (metabolite); stress metabolite with similar distributions of appearance with increases in cold condition occur in similar positions on the map).

Remarkable variations in terms of accumulation of various intracellular sugars and polyols were noticed through HPLC chromatogram (Additional file 1: Figure S1), when all the *Pseudomonas* cells were grown at 4 and 28°C (Table 2). The bacterial intracellular sugar content was expressed in terms of μg per mg of cell dry weight (μg mg^-1^ CDW^-1^). It was found that the accumulation of intracellular sugars varied in different psychrotrophic *Pseudomonas* strains at 4 and 28°C. Such changes were observed in the amount of glucose, trehalose, sucrose, mannitol and sorbitol. Figure 3 shows the grouping of stress metabolites based on their accumulation at two different temperatures for individual strain and indicates variation in accumulation of stress metabolites for each isolate. Most importantly, a prominent and statistically significant increase in intracellular raffinose was noticed during cold condition (at 4°C) in all the *Pseudomonas* isolates (Table 2, Figure 3). Accumulation of sucrose molecule at 4°C was found in *P. fluorescens* PPRs4, while, at 28°C it was not detected. Likewise, glucose molecule was not detected in bacterial growth at 4°C for all the isolates, while *P. lurida* NPRp15 showed noticeable amount of glucose at 28°C. Trehalose was accumulated in high amount in all the isolates at 4°C. *Pseudomonas* sp. PPERs23 accumulated significant amount of trehalose, mannitol and sorbitol, when grown at 28°C (Table 2). Accumulation of mannitol was found only in four isolates, interestingly three out of four isolates showed higher accumulation of mannitol at 28°C, *P. putida* PGRs4 showed higher accumulation of mannitol at 4°C (Table 2). Greater accumulation of sorbitol was noticed for NPRs3, PGRs4 and PGERs17 strains, when their cells were grown at 4°C in comparison to the cells grown at 28°C. Whereas, *Pseudomonas* sp. PPERs23 accumulated significant amount of sorbitol at 28°C.

**Table 2:** **Quantitative analysis of intracellular sugars and polyols content of** ***Pseudomonas*** **strains by HPLC**

***Pseudomonas*** strains	Temperature	Content (μg mg^-1^cell dry weight^-1^)*					
		Sugar’ content				Polyols content	
		Raffinose	Sucrose	Trehalose	Glucose	Mannitol	Sorbitol
***P. lurida*** **NPRs3**	**28°C**	8.92 ± 1.52a	-	-	-	2.73 ± 0.52a	-
	**4°C**	19.79 ± 2.26b	-	1.89 ± 0.21	-	2.23 ± 0.34a	9.36 ± 1.61
***P. lurida*** **NPRp15**	**28°C**	-	-	-	105.4 ± 3.9	43.23 ± 3.63	-
	**4°C**	41.69 ± 3.39b	-	3.19 ± 0.81	-	-	-
***Pseudomonas*** **sp. PPERs23**	**28°C**	3.18 ± 1.28a	-	16.46 ± 1.62b	-	7.12 ± 1.07b	22.06 ± 1.96b
	**4°C**	13.41 ± 1.76b	-	2.49 ± 0.48a	-	0.55 ± 0.01a	3.80 ± 1.56a
***P. putida*** **PGRs4**	**28°C**	4.38 ± 0.97a	-	0.44 ± 0.09a	-	-	3.17 ± 1.01a
	**4°C**	11.25 ± 1.13b	-	3.14 ± 0.84b	-	3.92 ± 0.66	50.49 ± 3.68b
***Pseudomonas*** **sp. PGERs17**	**28°C**	1.56 ± 0.54a	-	-	-	-	0.91 ± 0.03a
	**4°C**	9.90b	-	3.02 ± 1.6	-	-	4.32 ± 0.98b
***P. fluorescens*** **PPRs4**	**28°C**	6.76 ± 1.38	-	-	-	-	-
	**4°C**	14.22 ± 1.74	3.33 ± 1.25	-	-	-	-

*(-): Not detected.

Note: All values are mean of three (n = 3) experiments, followed by ± Standard deviation.

Letters (a,b) in the same column for each *Pseudomonas* strain indicate significant difference at 4°C and 28°C incubation temperatures.

### Accumulation of intracellular amino acids

The intracellular free amino acids’ concentrations in *Pseudomonas* strains were investigated under optimal (28°C) and low (4°C) temperature conditions. HPLC chromatograms of the *Pseudomonas* cells for 17 amino acids tested from their intracellular extract demonstrated significant variations at 4 and 28°C growth temperatures (Additional file 2: Figure S2 and Table 3). The intracellular amino acids’ expression pattern varied from strain to strain at 4 and 28°C, and the most prominent increase was observed in the concentrations of aspartic acid, proline and cysteine at 4°C (Table 3 and Figure 3). All *Pseudomonas* strains exhibited statistically significant increase in their aspartic acid (1.3 to 3.0 folds) and proline (1.1 to 2.8 folds) contents at 4°C. Significant accumulation of serine, arginine, threonine, cysteine, leucine and phenylalanine at 4°C was found among all the isolates, on the other hand reduction in concentrations of glycine, glutamic acid, lysine and methionine at 28°C was observed for most of the isolates. The *P. lurida* NPRs3 cells showed highest increase in concentration of aspartic acid, serine, glycine, arginine, and proline at 4°C, while no change was observed in lysine concentration. Substantial increase in accumulation of isoleucine (50.4 folds) and cysteine (5.6 folds) was noticed, whereas no changes were observed for glutamic acid and methionine for *P. lurida* NPRp15 cells grown at 4°C. Prominent increase in phenylalanine (24.2 folds) followed by histidine (4.6 folds), threonine (3.6 folds) and leucine (3.5 folds), and minor increase in tyrosine concentrations were observed at 4°C in *P. putida* PGRs4 cells (Table 3). For *Pseudomonas* sp. PGERs17 cells, enhanced concentrations were found for tyrosine (21.3 folds), threonine (4.2 folds), glutamic acid (3.9 folds), and proline (2.7 folds) at 4°C, while no changes were noticed for arginine (Table 3). Maximum increase was found for cysteine (46.5 folds) followed by leucine (7.4 fold), isoleucine (6.3 folds) and serine (4.5 folds) in *P. fluorescens* PPRs4 cells at 4°C (Table 3). However, low concentrations of glutamic acid, tyrosine, and lysine were found in the same cells at 4°C (Table 3).

**Table 3:** **Quantitative analysis (HPLC) of intracellular amino acids’ content of** ***Pseudomonas*** **strains at 4°C and 28°C growth temperatures**

Amino acid (pico mole mg^-1^cell dry wt.^-1^)*	***Pseudomonas*** strains											
	***P. lurida*** NPRs3		***P. lurida*** NPRp15		***Pseudomonas*** sp. PPERs23		***P. putida*** PGRs4		***Pseudomonas*** sp. PGERs17		***P. fluorescens*** PPRs4	
	At 4°C	At 28°C	At 4°C	At 28°C	At 4°C	At 28°C	At 4°C	At 28°C	At 4°C	At 28°C	At 4°C	At 28°C
**Aspartic acid**	65.8 ± 1.5b	39.9 ± 0.9a	33.1 ± 0.7b	20.3 ± 0.5a	14.1 ± 0.3b	11.1 ± 0.2a	333.9 ± 7.5b	166.5 ± 3.7a	51.7 ± 1.2b	27.5 ± 0.6a	18.0 ± 0.5b	5.9 ± 0.3a
**Proline**	4355 ± 98b	1997 ± 45a	7317 ± 164b	3751.2 ± 84a	1880 ± 42b	1229.7 ± 27a	1467 ± 33b	1352.2 ± 30a	4733.3 ± 106b	1738.2 ± 39a	1613 ± 26b	586.5 ± 13a
**Cysteine**	32.8 ± 0.7a	89.7 ± 2b	439.4 ± 9.9b	78.2 ± 1.8a	67.8 ± 1.5	-	89.9 ± 2b	52.5 ± 1.2a	30.8 ± 0.7b	17.4 ± 0.4a	71.8 ± 1.8b	1.5 ± 0.1a
**Serine**	485.0 ± 10.9b	57.9 ± 1.3a	28.1 ± 0.6b	23.0 ± 0.5a	12.0 ± 0.4a	21.6 ± 0.5b	14.2 ± 0.3b	7.7 ± 0.2a	13.6 ± 0.3a	19.0 ± 0.4b	13.1 ± 0.6b	2.9 ± 0.1a
**Glutamic acid**	11.8 ± 0.3a	26.2 ± 0.6b	14.8 ± 0.3a	16.1 ± 0.4a	8.8 ± 0.2b	3.9 ± 0.1a	26.1 ± 0.6a	46.5 ± 1.1b	28.7 ± 0.6b	7.4 ± 0.2a	5.9 ± 0.3a	8.0 ± 0.4b
**Glycine**	662.0 ± 14.9b	347.5 ± 7.8a	274.5 ± 6.2a	370.7 ± 8.3b	350.8 ± 7.9a	184.7 ± 4.2b	137.7 ± 3.1b	121.5 ± 2.7a	351.2 ± 7.9a	533.3 ± 12b	288.8 ± 6.2b	272.9 ± 6.1a
**Histidine**	97.6 ± 2.2a	192.6 ± 4.3b	148.2 ± 3.3b	134.4 ± 3a	50.8 ± 1.1a	63.0 ± 1.4b	20.9 ± 0.5b	4.6 ± 0.1a	103.9 ± 2.3b	55.6 ± 1.3a	73.9 ± 1.8b	65.9 ± 1.5a
**Arginine**	158.1 ± 3.6b	27.6 ± 0.6a	12.9 ± 0.3b	9.8 ± 0.2a	4.5 ± 0.1b	1.2 ± 0.1a	58.2 ± 1.3a	81.1 ± 1.8b	8.2 ± 0.2a	9.4 ± 0.2a	7.7 ± 0.3b	4.4 ± 0.1a
**Threonine**	190.5 ± 4.3a	1084.3 ± 24b	9194.7 ± 20b	3757.1 ± 84a	5143.0 ± 115a	6722.1 ± 151b	6510.4 ± 146b	1795 ± 40.4a	3003.8 ± 67.6b	714.0 ± 16a	6479.2 ± 57b	3890.4 ± 87a
**Alanine**	57.5 ± 1.3a	167.73.8b	107.9 ± 2.4b	94.1 ± 2.1a	38.3 ± 0.9a	39.7 ± 0.9a	104.6 ± 2.4a	890.9 ± 20b	91.0 ± 2b	66.3 ± 1.5b	46.5 ± 1.3a	50.1 ± 1.1b
**Tyrosine**	30.2 ± 0.7a	46.5 ± 1.2b	39.2 ± 0.9b	19.6 ± 0.4a	10.7 ± 0.3a	9.5 ± 0.4a	6.5 ± 0.1a	3874.7 ± 87b	16.8 ± 0.4b	0.8a	14.0 ± 0.5a	166.8 ± 3.8b
**Valine**	27.1 ± 0.6a	64.9 ± 1.5b	40.0 ± 1.9b	23.2 ± 0.5a	9.5 ± 0.2a	8.3 ± 0.3a	3.1 ± 0.1a	26.4 ± 0.6b	17.0 ± 0.4b	6.5 ± 0.1a	14.1 ± 0.4b	7.0 ± 0.2a
**Methionine**	11.6 ± 0.3a	37.50.8b	18.5 ± 0.4a	18.9 ± 0.4a	5.5 ± 0.1a	7.3 ± 0.2b	13.2 ± 0.3b	10.0 ± 0.2a	12.8 ± 0.3a	11.4 ± 0.3a	10.3 ± 0.3a	10.2 ± 0.2a
**Lysine**	40.0 ± 0.9a	41.6 ± 0.9a	58.5 ± 1.3a	153.7 ± 3.5b	9.8 ± 0.5a	20.2 ± 0.5b	303.5 ± 6.8a	301.2 ± 6.8a	69.1 ± 1.6a	120.9 ± 2.7b	5.1 ± 0.2a	11.3 ± 0.3b
**Isoleucine**	16.3 ± 0.4a	54.4 ± 1.2b	778.3 ± 17.5b	15.4 ± 0.3a	0.2a	0.4a	49.8 ± 1.1b	27.9 ± 0.6a	-	-	34.5 ± 0.7b	5.5 ± 0.2a
**Leucine**	9.7 ± 0.2a	18.5 ± 0.4b	103.6 ± 2.3b	12.3 ± 0.4a	6.1 ± 0.2a	5.1 ± 0.1a	78.9 ± 1.8b	22.6 ± 0.5a	9.1 ± 0.3b	6.7 ± 0.2a	73.1 ± 1.3b	9.9 ± 0.3a
**Phenylalanine**	21.2 ± 0.5a	81.6 ± 1.8b	38.6 ± 0.9b	23.9 ± 0.5a	6.6 ± 0.3a	5.4 ± 0.2a	118.9 ± 2.7b	4.9 ± 0.1a	8.3 ± 0.2b	4.9 ± 0.1a	130.1 ± 3.3b	5.4 ± 0.1a

*(-): Not detected.

Note: All values are mean of three (n = 3) experiments, followed by ± Standard deviation.

Letters (a,b) in the same column for each *Pseudomonas* strain indicate significant difference at 4°C and 28°C incubation temperatures.

### Stastistical analysis

Correlation analysis proved existence of significant relationship between the measured cold stress parameters and the bacterial growth conditions (i.e., temperature: coordinate). The PCA for correlation of individual isolate has been shown in Figure 3. The first two factorial axes represent 94.7 to 97.14% variance in the data. Except *Pseudomonas* NPRs3, factor F1 represented the grouping of stress metabolites and reflected their substantial accumulation at 4°C. Factor F2 represented the grouping of stress metabolites accumulated maximally at 28°C. Maximum stress metabolites were accumulated at 4°C for two *Pseudomonas* isolates, i.e., NPRp15 and PPRs4 (Figure 3[b,f]). The parameters placed at a strong positive side of the factor F1 were highly correlated with the cold temperature and indicated higher accumulation at cold temperature. However, the parameters which were placed at negative side of the factor F1 showed negative correlation with the cold temperature and indicated less or nil production at cold temperature. Similarly, the parameters which were placed at the strong positive side of the axis of factor F2 indicated minor role in cold adaptation and showed higher accumulation at optimum growth temperature of 28°C. The parameters placed at the negative side of the factor F2 showed less accumulation and less activity at 28°C, and high correlation with cold temperature. Only freezing survival parameter was found on the negative side of the factor F2 for five *Pseudomonas* strains with exception of NPRs3 cells (Figure 3[a]). The parameters which were grouped together in the PCA plot showed high correlation, whereas parameters which were grouped in opposite direction indicated the negative correlation. The parameters placed at the middle of the PCA model reflected no correlation with the factors (i.e., growth temperatures) and showed either equal or nil accumulation. These include no accumulation of glucose and sucrose for five strains except NPRp and PPRs4, respectively (Figure 3[b,f]).

**Figure 4:**
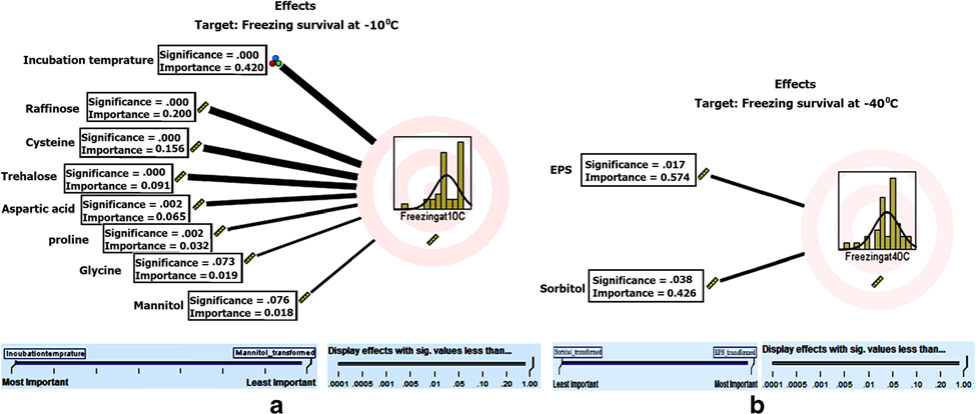
**The combined analysis of relationship/effect of stress metabolite with/on freezing survival [(4a) -10 degree C; (4b) -40 degree C] of** ***Pseudomonas*** **strains [automated linear model (95% CI)].**

The relationship/effect of measured cold stress metabolites on bacterial freezing survival at -10 and -40°C was evaluated using automated linear modeling (ALM) analysis (Figure 4). The model contained only important predictor(s) (i.e., stress metabolites) for freezing survival. Both the models were found statistically significant (p < 0.05) with 70 to 75% accuracy. Six cold stress parameters were identified as important predictors for freeze survival at -10°C (Figure 4[a]). The incubation temperature showed significant correlation (p = 0.000) with freeze survival at -10°C and demonstrated important role (42%) in bacterial freeze survival. Bacterial survival to freezing conditions was paralleled by an increase in the intracellular raffinose level and showed significant association (p = 0.000) with freezing survival at -10°C, and also suggested that raffinose contribute in bacterial freeze survival. Likewise, high accumulation of cysteine, trehalose, aspartic acid and proline showed positive relationship with bacterial high freezing survival at -10°C with 15.6, 9.1, 6.5 and 3.2% contribution, respectively. EPS and sorbitol were identified as most important stress metabolites (predictors) for bacterial freeze survival at -40°C (Figure 4[b]).

## Discussion

The Himalayan region provides an opportunity to obtain microbes that have experienced extended exposure to cold temperatures, reduced water activities, radiation and low nutrient accessibility. The cold adaptation related properties of psychrotrophic *Pseudomonas* cells showed high cellular metabolism activities in cold conditions (Mishra et al. 2008; 2009; 2011). It has been well established that the cold-active enzymes and efficient growth rates are used to facilitate and maintain the adequate metabolic fluxes at near freezing temperature for cold-adaptation (Shivaji and Prakash 2010). The great metabolic flexibility of *Pseudomonas* species allows them to inhabit diverse environments and capable of a high level environmental ubiquity even in cold/freezing habitats (Timmis 2002; Remold et al. 2011; Cray et al. 2013b). This study clearly demonstrates that cold acclimatized cells (grown at 4°C) had higher freezing-thawing survival over non-acclimatized cells (grown at 28°C), and linear modeling analysis clearly proved that the incubation temperature was the main factor for Pseudomonads’ freezing survival. The overall freezing-thawing survival of *Pseudomonas* strains at -10°C was found be 98% (Additional file 3: Table S1). This feature of Pseudomonads suggests their survival persistence in Himalayan extreme freezing-thawing conditions during winter season. One of the major freezing survival strategy might be the evolution of strains that are capable of utilizing a large number of carbon sources (Ponder et al. 2005). This might be more relevant because the alpine environment is highly heterogeneous with pockets of specific carbon compounds, a large number of bacterial strains that have recently gained or lost the ability to grow on a particular source of carbon may exist and supported the adaptability, versatility and environmental ubiquity, and prevalence of *Pseudomonas* genus in the Himalayan region (Mishra et al. 2010; Remold et al. 2011; Cray et al. 2013b).

Ice crystal formation is the primary risk associated with the freezing-thawing of microbial cells and leads to membrane damage, and parallels the situation of dehydration/desiccation of the cells. Crowe et al. (1984) reported that the rehydration condition causes most damage to the cells. At low temperatures (both above and below 0°C) the intracellular environment usually becomes dehydrated and this increases solute concentration as well as free radical formation. As a result cold and solute-stress are to a large extent inseparable (Chin et al. 2010). A key component of cryotolerance in bacterial cells is tolerance to desiccation, solute-induced stress, and oxidative stress (Hallsworth et al. 2003; Bhaganna et al. 2010; Gülez et al. 2012; Pablo et al. 2013; Jonathan et al. 2013). Lowering of ice nucleation temperature and controlling the freezing temperature and shape of the ice crystal have been identified as two possible strategies for microbial cells to avoid freezing conditions (Kawahara et al. 1991). Whereas development of freezing tolerance by producing cryoprotectant compounds or adaptation of cytoplasmic enzymes to cold conditions for protecting cytoplasmic components is the third strategy used by microbial cells to survive in freezing conditions as these molecules depress freezing point for the protection of cells (Yamashita et al. 2002). In the present study, all *Pseudomonas* strains didn’t display the presence of type-I and/or type-II ice nuclei as found in ‘ice plus’ bacteria. Thus, all the collected strains were considered as ‘ice minus’ bacteria because they lack Ina proteins present on bacterial cell wall that act as a nucleation centre for ice crystals, which are mostly active in between -2 to -7°C. All *Pseudomonas* strains demonstrated very low level of type-III ice nuclei which is typically active between -7 to -10°C. Presentation of less ice nuclei by the Pseudomonads indicates their freeze survival strategy by lowering the ice nucleating temperature. Therefore, it can be suggested that low ice nucleation activity of Pseudomonads makes them capable of inhibiting the ice formation, which might required for freezing survival.

Microbes produce EPS, which is stored as a thick gel surrounding the cell, the major ecological characteristic of EPS is that it can form and maintain protective microhabitats around microorganism in aquatic and frozen atmosphere (Stoderegger and Herndl 1998; Decho 1990; Tamaru et al. 2005). We found that EPS production by the *Pseudomonas* strains was higher at lower temperatures (4 or 15°C) in comparison to their optimal growth temperature (28°C). Enhanced EPS production by the Pseudomonads at low temperature suggested that EPS plays an important role in desiccation protection or prevention of drying of bacterial cells from freezing temperature (Figures 2 and 4; Roberson and Firestone 1992). The production of EPS is associated with the biofilm formation. The fact that the Himalayan strains overproduce EPS at low temperature might suggests that under these conditions, the *Pseudomonas* strains show higher biofilm formation, and even the process of root colonization enhances at low temperature (Mishra et al. 2011).

Studies of low temperature tolerance in microbial cells demonstrated that the flexibility of cellular macromolecules can be the limiting factor/failure-point for growth windows at low temperatures, and showed that a chaperonin (chao- and kosmotrop) can extend the biotic window for growth down to lower temperatures (Margesin and Schinner 1999; Ferrer et al. 2003). Hence, it can be assumed that the collected Pseudomonads were also following the third type cold evading strategy to thrive in freezing conditions by synthesizing various chaperonin/cryoprotectants, i.e., sugars, polyols and amino acids, in order to protect their cytoplasmic components. These cryoprotectants are known to depress freezing point to evade crystallization (Chattopadhyay 2002).

Raffinose, like other sugars plays a cryoprotective role by interacting with membrane lipids and proteins and decreases the risk of intracellular ice-crystal formation that causes cellular osmotic dehydration during cryopreservation (Agca et al. 2002; Tuncer et al. 2010). The effect of raffinose related to oxidative stress has been considered as an indirect effect of sugar signaling and triggers the production of specific reactive oxygen species (ROS), such as, hydroxyl radicals’ scavengers (Van-den Ende and Valluro 2009). Though, the role of raffinose has been defined in recent years in alleviation of oxidative stress and as a cryoprotectant (Van-den Ende and Valluro 2009; Tuncer et al. 2010), but, still no reports are available related to the accumulation of raffinose in bacterial cells in response to cold stress conditions. Here, we found high accumulation of intracellular raffinose content in all the tested Pseudomonads in response to their growth at 4°C prior to freezing at -10 and/or -40°C, and it was further confirmed through linear modeling analysis and supported its role in bacterial freeze survival (Figure 4). Likewise trehalose, raffinose is a kosmotrophic substance that has a stabilizing effect on macromolecular structure (Cray et al. 2013a). Therefore, it may possible or it can be hypothesized that the protective effect of raffinose as observed in current study of Himalayan Pseudomonads utilizes a different mechenism from that of glycerol and fructose (Chin et al. 2010).

The significance of chao- and kosmotropicity for the maintenance of structure and activities of macromolecular systems have been well characterized *in*-*vitro*, whereas, the degree to which they facilitate and/or limit the activities of cellular macromolecules *in-vivo* remains relatively unclear (Duda et al. 2004; Chin et al. 2010; Cray et al. 2013a). Nevertheless, it has been established that chaotropicity-mediated stresses elicit specific stress responses in microbial cells (Bhaganna et al. 2010). Chaotropicity has been shown to not only limit life processes but can render potential environmental habitats (Hallsworth et al. 2007; Cray et al. 2013a). The kosmotropicity nature of trehalose (non-reducing disaccharide) plays an important role in developing the ability of organisms to resist against adverse environmental conditions (Kandror et al. 2002; Cray et al. 2013a). Trehalose stabilizes the membrane and proteins by replacing water and preserves the intracellular water structure (Sano et al. 1999). High intracellular trehalose accumulation was found in all *Pseudomonas* strains except *P. fluorescence* PPRs4 cells when they grown at 4°C prior to freezing and the same was supported by linear modeling analysis (Figure 4). Our findings were in congruence with earlier studies where accumulation of higher trehalose was related with its cryoprotectant function (Kaasen et al. 1992; Mitta et al. 1997). Regarding *P. fluorescens* PPRs4, we can speculate that trehalose might replaced by sucrose and plays similar role of cryoprotectant, as higher sucrose accumulation was noticed in the said strain (Cray et al. 2013b).

The cryoprotectant property of glucose has been previously documented by Koda et al. (2002). On the similar lines, we found that *P. lurida* NPRp15 cells accumulated higher glucose content when grown at 28°C and also demonstrated freezing survival at -10°C. Hence, it can be suggested that glucose plays a significant role in cryoprotection of microbial cells. Two more kosmotropic solutes mannitol and sorbitol act as stress protectants and has been previously investigated (Chatuverdi et al. 1997; Kets et al. 1996; Bhaganna et al. 2010). The principle role of mannitol for the de novo-synthesized polyol mannitol in osmoadaptation of a heterotrophic *P. putida* has been discovered recently (Bhaganna et al. 2010). Earlier studies reported that mannitol accumulation increases in microbial cells under various stress treatments, like, heat, salt and/or their combination (Managbanag and Torzilli 2002; Chatuverdi et al. 1997). Analogous to above, we also noticed enhanced accumulation of intracellular mannitol and sorbitol in all the *Pseudomonas* strains except PPRs4 strain grown at 4 and 28°C, and it can be assumed that glycerol might replaced by mannitol/sorbitol in these *Pseudomonas* strains as we failed to detect glycerol in bacterial cells.

Moreover, the collected cold tolerant *Pseudomonas* strains were found to protect cytoplasmic components by synthesizing specific free amino acids needed for freezing survival and cold adaptation of the microbial cells. These amino acids act as chemical chaperones which prevent the aggregation of cellular proteins during stress conditions and their possible function is to regulate the fluidity of membrane at lower temperatures (Chattopadhyay and Jagannadham 2001; Chattopadhyay 2002; Ferrer et al. 2003; Bhaganna et al. 2010; Jonathan et al. 2013). Enhanced production of intracellular proline has been reported in microorganisms in order to improve their freeze tolerance and osmotic stress (Morita et al. 2003; Jonathan et al. 2013; Kempf and Bremer 1998). This indicates that proline accumulation might be a general protective strategy against freeze stress evasion. Additionally, the intracellular accumulation of charged amino acids, viz., arginine, aspartic acid and glutamate also seems to enhance microbial freeze tolerance and act as cryoprotectants (Shima et al. 2003; Jenkelunas 2013). These amino acids thought to play important roles as general acids in enzyme active centers, as well as in maintaining the solubility and ionic character of proteins (Shima et al. 2003; Jenkelunas 2013). In view of previous reports, high accumulation of intracellular proline, arginine and glutamate in collected Pseudomonads suggests their cryoprotective role for freezing survival. Cysteine and methionine are sulphur-containing amino acids. Cysteine is a powerful antioxidant and can react with itself to form an oxidized dimer by forming a disulfide bond. The environment within a cell is too reducing for disulfides to form, but in the extracellular environment, disulfides can form and play a key role in stabilizing many proteins (Sen 2005; Ladenstein and Ren 2008). Disulfide bonds are important for protection of bacteria as a reversible switch that turns a protein on or off when bacterial cells are exposed to oxidation reactions. Hydrogen peroxide (H_2_O_2_) in particular could severely damage DNA and kill the bacterium at low concentrations if not for the protective action of the SS-bond (Ladenstein and Ren 2008). Likewise, methionine mostly acts as a precursor amino acid for glutathione. It plays an important role in the antioxidant defense mechanism by reacting readily with oxidants to form methionine sulfoxide (Livine et al. 1999). The present study suggests that high intracellular cysteine and methionine synthesis in collected Pseudomonads grown at 4°C might be an integral part of cold survival strategy to avoid damages from oxidative stress during cold conditions.

## Conclusions

In conclusion, it is the physicochemical diversity of stress protectants produced by Pseudomonads that confer their remarkable tenacity and stress tolerance. Each type of compatible solute/cryoprotectant has protective effects via different mechanisms. Accumulation of diverse amino acids, sugars and polyols (including EPS) under cold stress are important characteristics of Himalayan psychrotrophic *Pseudomonas* strains. The most novel and intriguing finding of this study was, intrecellular accumulation of raffinose, cysteine and aspartic acid in bacterial cells as a key metabolites at low temperature. The characterization of these traits are potentially important for beginning to understand these adaptations in microbial community present in Himalayan region. The present findings are part of unfolded field of stress biology, and it will surely have implications for the studies related to microbial diversity present in extreme conditions of high altitudes.

## Materials and methods

### Bacterial culture conditions and chemicals

The psychrotrophic *Pseudomonas* strains used in the present study were previously isolated from different plant root zones that were collected from the high altitude regions of NW Himalaya (Mishra et al. 2011; Bisht et al. 2013). Bacterial cultures were maintained on Nutrient Agar (NA) and Kings B slants, respectively, and preserved in 60% glycerol at -80°C. The submission details of all the six *Pseudomonas* strains and their growth curve studies (at three different temperatures, i.e., 4°C, 15°C and 28°C) have been published earlier (Mishra et al. 2008; 2009; 2011). All reagents were of analytical grade and procured from Merck, Sigma Aldrich, HiMedia Laboratories.

### Assessment of survival after freezing-thawing

Six bacterial cell samples (6 strains × 3 replicates = 18) from each *Pseudomonas* strain were prepared and each set was grown separately into 5 ml LB medium at two different temperatures 4 and 28°C (48 h incubation period for 4°C culture and 24 h for 28°C culture). Both cultures (4 and 28°C) were kept separately at preset temperature of -10 and -40°C temperature for 48, 72 and 96 h. Bacterial cultures were thawed at room temperature, appropriate dilutions were plated and incubated at 28°C for 48 h and CFU counts were measured for 48, 72 and 96 h of freeze shifted bacterial cultures. Freeze-thaw survival of *Pseudomonas* spp. was determined at -10 and -40°C by comparing the log CFU counts before and after the freezing treatment. All experiments were performed in duplicates.

### Quantification of exopolysaccharide (EPS) production

Bacterial cells were grown in 100 ml Kings B and nutrient broth at three different temperatures, i.e., 4, 15 and 28°C for 48 h. Following the incubation, bacterial cells were harvested and EPS was extracted following the method of Underwood et al. (1995). Precisely, the bacterial cells were centrifuged at 10,000 rpm (Sigma Model 2 K15, Rotor No. 12132) at 4°C for 15 min. The cell pellet was washed twice with sterile distilled water, treated with 10 mM EDTA (w/v), vortexed for 15 min, and finally recentrifuged at 10,000 rpm for 20 min at 4°C to extract the cell-bound EPS. Extraction process was repeated and EPS samples were pooled and precipitated using chilled acetone and centrifuged at 10,000 rpm for 10 min. The cell pellet was collected and their dry weight was measured.

### Determination of ice nucleation activity

Ice nucleation activities (INA) [ice nuclei per colony-forming-unit (CFU)] of potential cold tolerant *Pseudomonas* strains were measured by freeze-drop method (Vali 1971; Lindow 1990). Bacterial cultures were grown into 100 ml Luria broth (LB) medium at 4°C for 24 to 96 h. One ml of culture was centrifuged at 6,000 rpm for 5 min at 4°C and collected pellet was washed twice with 0.85% phosphate buffer saline (PBS; pH 7.2; w/v). The cell pellet was resuspended into 1.0 ml of phosphate buffer (pH 7.2) and vortexed vigorously. Nearly 10 μl (equivalent to 30 drops) of the cell suspension was placed on a parafilm coated aluminum boat floating on an ethanol bath at preset temperature of -5 and -10°C. The number of frozen droplets were counted after 2, 5, 15 and 20 min, respectively and bacterial concentration was measured by plating of serial dilution of bacterial cells on Kings B medium followed by incubation for 48 h. The ice nucleation activity was calculated and expressed in log ice nuclei CFU^-1^ (Vali 1971; Lindow 1990).

### Preparation of intracellular cell extract for detection of cryoprotectants

Two sets (6 × 2 = 12) of each bacterial isolate was prepared and grown separately into 100 ml LB broth medium at 4 and 28°C for 36 h. Afterwards, the cultures were centrifuged at 5°C for 10 min at 8,000 rpm and resultant cell pellets were washed three times with 0.85% PBS (pH 7.2). The cell pellets were resuspended into 1.0 ml of phosphate buffer (pH 7.2) and disrupted by sonication (Soni Prep 150, Sanyo) in 3 cycles at 8 μm (amplitude) for 2 min with 45 sec cooling interval. The cell debris of each culture was removed by centrifugation at 10,000 for 15 min at 4°C. The supernatant was filtered (0.22 μm) and samples were stored at -20°C. Standard sugar solutions of specific concentration (100, 200, 300, 400 and 500 mg l^-1^) were prepared in phosphate buffer (pH 7.0). This experimental part was performed in duplicate.

### Analysis of intracellular sugars

High performance liquid chromatography (HPLC) (Waters Corporation, USA) analysis was employed to analyze the intracellular sugars. The HPLC system consisted of an isocratic pump (Waters, 600 Delta), DES-1008D interface (D-Link, China), Waters temperature controller model TC2 and evaporative light scattering detector (Waters 2424 ELSD) controlled by the ‘Empower’ program. Waters Spherisorb 5 μm NH_2_ (250 × 4.6 mm) chromatographic column was used during the analysis. Ten μl of the sample was injected into the Spherisorb column and separated with the mobile phase (67% acetonitrile and 33% water) at a flow rate of 1 ml min^-1^. The chromatographic parameters, like, gas pressure, detector gain value, column temperature, run time and nebulizer tube temperature were 50 psi, 10, 25°C, 30 min and 60°C, respectively. Various sugars, like, D-Xylose, D-Glucose, D-Sorbitol, Trehalose and Raffinose were quantified using external standard method, and samples were analyzed in triplicate.

### Analysis of intracellular free amino acids

Amino acid standard H-kit, amino acid solvent-A (Aqueous buffer, Waters AccQ•Tag™), derivatization regent that contain AccQ•Fluro Borate buffer and AccQ•Fluro reagent were procured from Waters Corporation, USA. Acetonitrile and deionised water were used as solvent-B. Samples were prepared according to the manufacturer’s instruction. The chromatographic analysis was performed on HPLC system as mentioned in the upper section using multi ƛ fluorescence detector (Waters 2475) attached with photodiode array detector (Waters 2996). Ten μl sample was injected in a Waters AccQ•Tag™ (Waters, Ireland) column and seprated by mobile phase (60% acetonitrile and 1/10^th^ concentration of Waters AccQ•Tag™ buffer) at gradient flow rate of 1 ml min^-1^. The chromatographic parameters, like, detector gain value, column temperature, and run time were maintained as 10, 25°C and 65 min, respectively. Intracellular free amino acids were quantified by measuring peak area using the external standard method and samples were analyzed in triplicate.

### Stastistical analysis

Descriptive statistics was employed to represent the means and standard deviations. Student’s *t*-test was used to compare the mean values at 4 and 28°C for stress metabolites. In order to investigate the expression pattern of stress metabolites for individual isolate and its correlation with growth temperatures (4 and 28°C), Principle component analysis (PCA) was performed for all cold stress related measured parameters along with the growth temperature. PCA analysis was carried out using the XLSTAT (version 2013) program. Additionally, to predict the relationship and effect of cold stress metabolites on bacterial freezing survival at -10 and -40°C, an automated linear modeling (ALM), using forward stepwise with information criterion (standard model) was performed. For the modeling, data gathered during bacterial growth at both the temperatures (4 and 28°C) were combined and analyzed using SPSS program. The linear modeling was performed with the goal of selection of the most explanatory model that can explain the relationship and effect between independent (stress metabolite) and dependent (freezing survival) variables.

## Electronic supplementary material

Additional file 1: Figure S1a: HPLC chromatogram of intracellular sugars’ and polyols’ contents of *Pseudomonas* strains grown at 4 and 28°C. **Figure S1b.** HPLC chromatogram of intracellular sugars and polyols’ contents of *Pseudomonas lurida* NPRp15 Grown at 4°C and 28°C. **Figure S1c.** HPLC chromatogram of intracellular sugars and polyols’ contents of *Pseudomonas* sp. PPERs23 grown at 4°C and 28°C. **Figure S1d.** HPLC chromatogram of intracellular sugars and polyols’ contents of *Pseudomonas putida* PGRs4 grown at 4°C and 28°C. **Figure S1e.** HPLC chromatogram of intracellular sugars and polyols’ contents of *Pseudomonas* sp. PGERs17 grown at 4°C and 28°C. (PPTX 1 MB)

Additional file 2: Figure S2a: HPLC chromatogram of intracellular amino acids’ contents of *Pseudomonas* strains grown at 4 and 28°C. **Figure S2b.** HPLC chromatogram of intracellular amino acid contents of *Pseudomonas lurida* NPRp15 grown at 4°C and 28°C. **Figure S2c.** HPLC chromatogram of intracellular amino acid contents of *Pseudomonas* sp. PPERs23 grown at 4°C and 28°C. **Figure S2d.** HPLC chromatogram of intracellular amino acid contents of *Pseudomonas putida* PGRs4 grown at 4°C and 28°C. **Figure S2e.** HPLC chromatogram of intracellular amino acid contents of *Pseudomonas* sp. PGERs17 grown at 4°C and 28°C. **Figure S2f.** HPLC chromatogram of intracellular amino acid contents of *Pseudomonas fluorescens* PPRs4 grown at 4°C and 28°C. (PPTX 1 MB)

Additional file 3: Table S1: Comparative analysis of stress metabolites accumulation/production at cold (4°C) and optimum growth temperature (28°C) for all six *Pseudomonas* strains (combined average of all strains). (DOCX 17 KB)
