# Supplementary material for: Cryotolerance strategies of Pseudomonads isolated from the rhizosphere of Himalayan plants
Source: Springerplus. 2013 Dec 12;2:667. doi: 10.1186/2193-1801-2-667 (PMC3868706; doi:10.1186/2193-1801-2-667)
Supplement: Supplementary file 2 — Additional file 2: Figure S2a: HPLC chromatogram of intracellular amino acids’ contents of Pseudomonas strains grown at 4 and 28°C. Figure S2b. HPLC chromatogram of intracellular amino acid contents of Pseudomonas lurida NPRp15 grown at 4°C and 28°C. Figure S2c. HPLC chromatogram of intracellular amino acid contents of Pseudomonas sp. PPERs23 grown at 4°C and 28°C. Figure S2d. HPLC chromatogram of intracellular amino acid contents of Pseudomonas putida PGRs4 grown at 4°C and 28°C. Figure S2e. HPLC chromatogram of intracellular amino acid contents of Pseudomonas sp. PGERs17 grown at 4°C and 28°C. Figure S2f. HPLC chromatogram of intracellular amino acid contents of Pseudomonas fluorescens PPRs4 grown at 4°C and 28°C. (PPTX 1 MB) [file 40064_2013_735_MOESM2_ESM.pptx]

## Slide 1
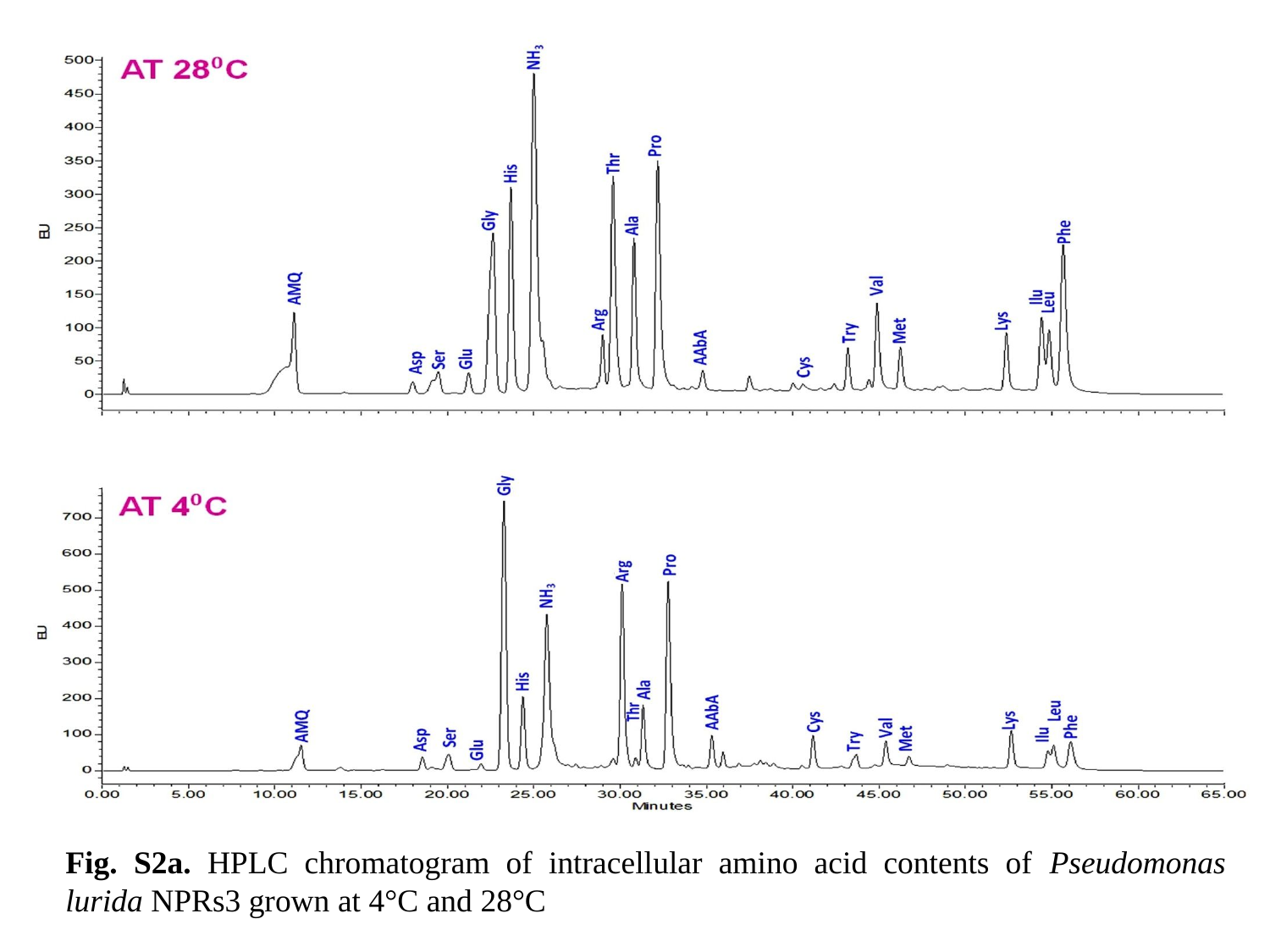

Fig. S2a. HPLC chromatogram of intracellular amino acid contents of Pseudomonas lurida NPRs3 grown at 4°C and 28°C

## Slide 2
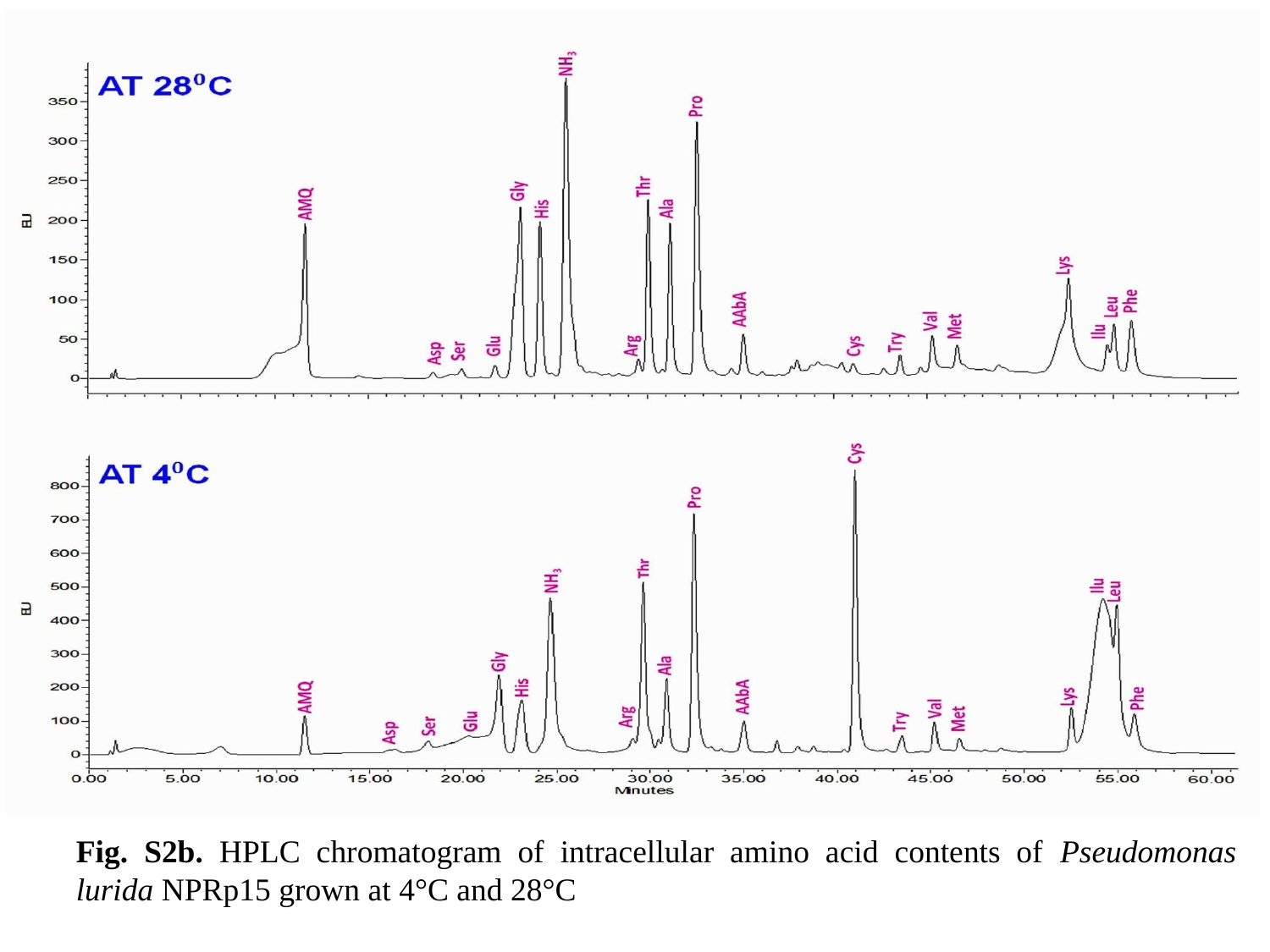

Fig. S2b. HPLC chromatogram of intracellular amino acid contents of Pseudomonas lurida NPRp15 grown at 4°C and 28°C

## Slide 3
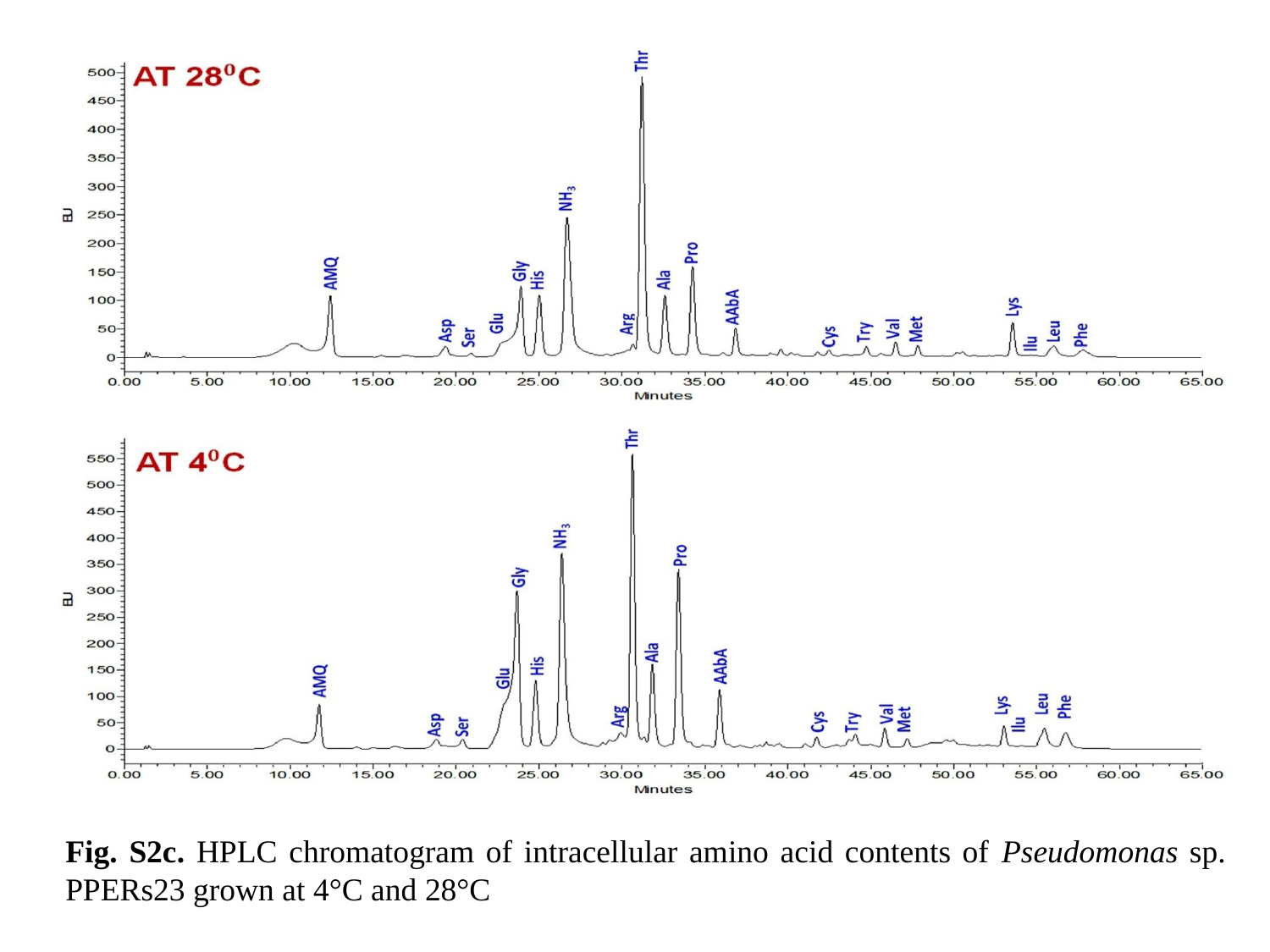

Fig. S2c. HPLC chromatogram of intracellular amino acid contents of Pseudomonas sp. PPERs23 grown at 4°C and 28°C

## Slide 4
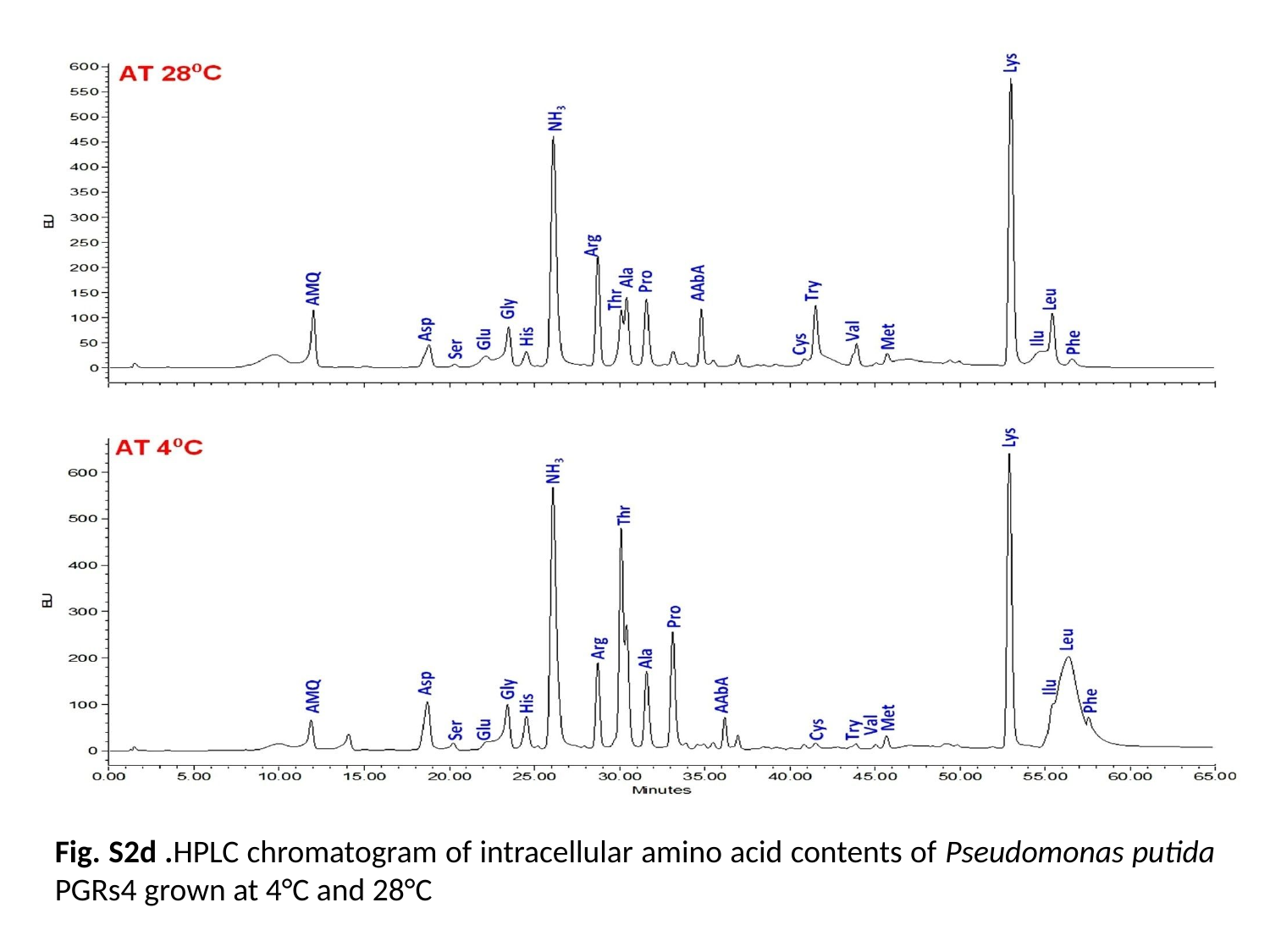

Fig. S2d .HPLC chromatogram of intracellular amino acid contents of Pseudomonas putida PGRs4 grown at 4°C and 28°C

## Slide 5
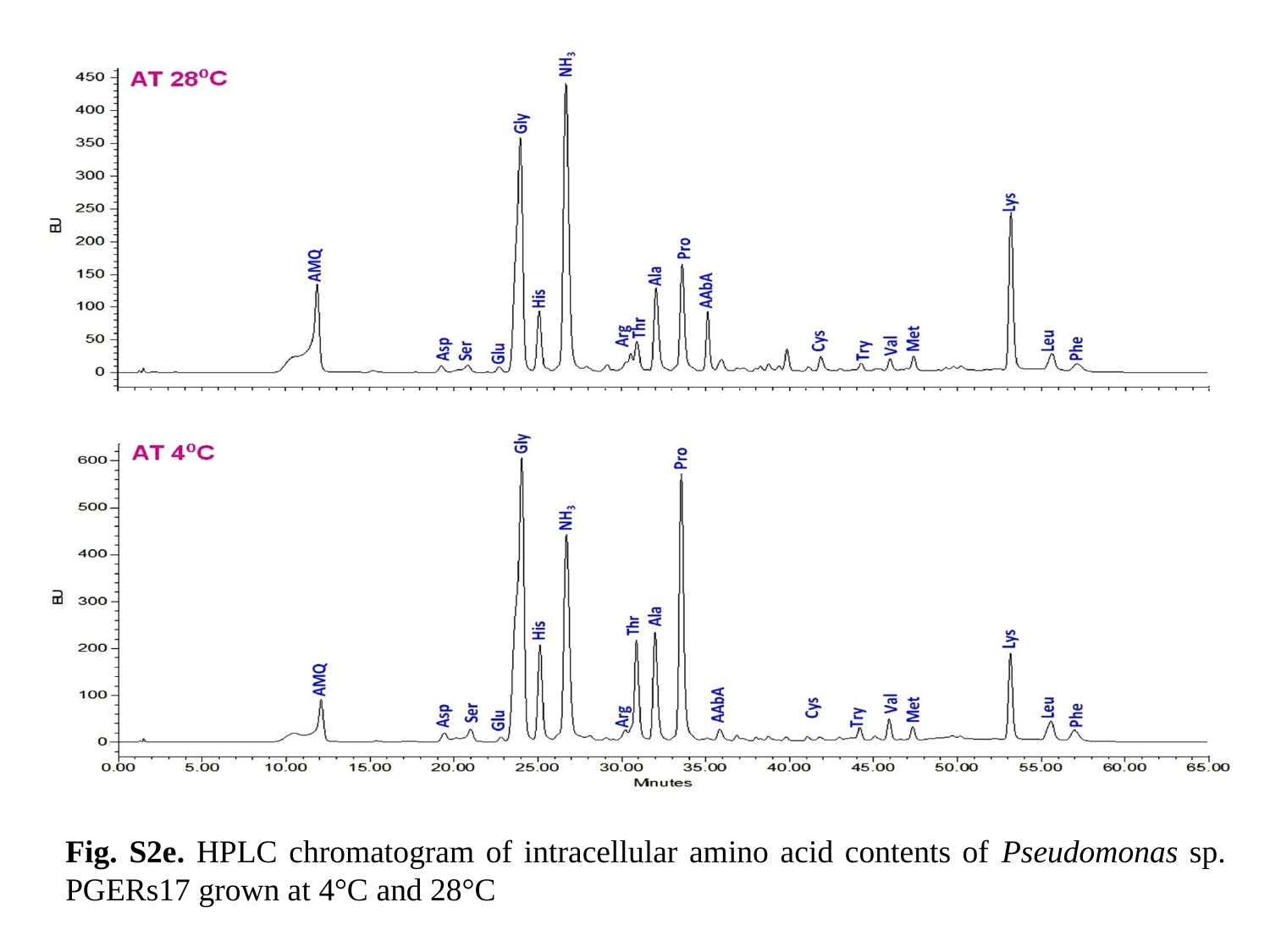

Fig. S2e. HPLC chromatogram of intracellular amino acid contents of Pseudomonas sp. PGERs17 grown at 4°C and 28°C

## Slide 6
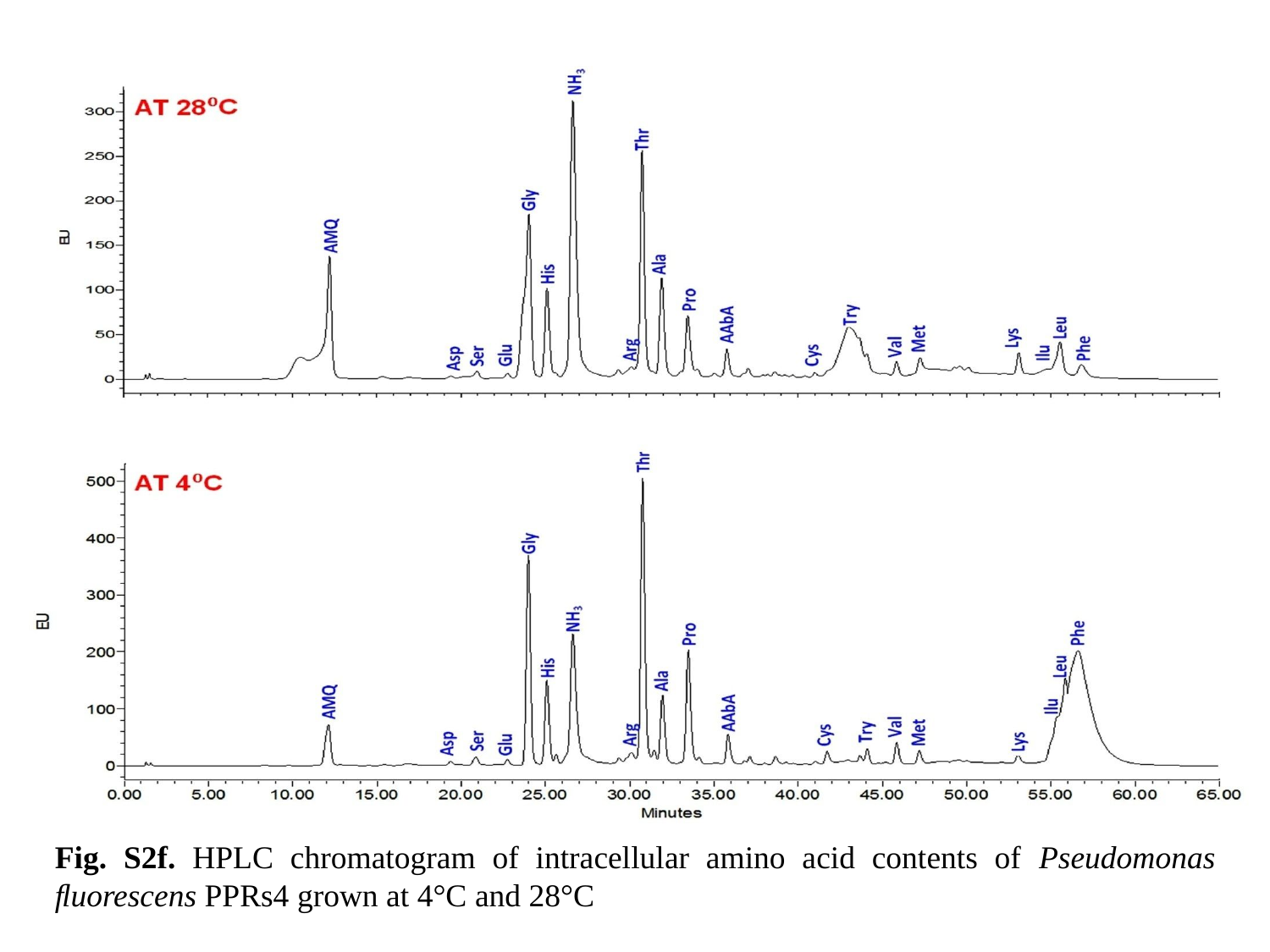

Fig. S2f. HPLC chromatogram of intracellular amino acid contents of Pseudomonas ﬂuorescens PPRs4 grown at 4°C and 28°C
