# Supplementary material for: Cryotolerance strategies of Pseudomonads isolated from the rhizosphere of Himalayan plants
Source: Springerplus. 2013 Dec 12;2:667. doi: 10.1186/2193-1801-2-667 (PMC3868706; doi:10.1186/2193-1801-2-667)
Supplement: Supplementary file 3 — Additional file 3: Table S1: Comparative analysis of stress metabolites accumulation/production at cold (4°C) and optimum growth temperature (28°C) for all six Pseudomonas strains (combined average of all strains). (DOCX 17 KB) [file 40064_2013_735_MOESM3_ESM.docx]

**Table S1.** Comparative analysis of stress metabolites accumulation /production at cold (4⁰C) and optimum growth temperature (28⁰C) for all six *Pseudomonas* strains (combined mean of all strains)

| **Higher expression at cold temperature (mean)** | | | **Higher expression at optimum temperature (mean)** | | |
| --- | --- | --- | --- | --- | --- |
| **Characteristics** | Incubation temperature | | **Characteristics** | Incubation temperature | |
|  | **28⁰C** | **4⁰C** |  | **28⁰C** | **4⁰C** |
| Freezing survival at -10⁰C | 92.84a | 97.98b | Glucose | 17.95 | 0.00 |
| Freezing survival at -40⁰C | 73.35a | 81.40b | Trehalose | 2.88a | 2.34a |
| EPS | 5.36a | 11.06b | Mannitol | 9.04b | 1.14a |
| Sucrose | 0.00 | 0.57 | Glutamic acid | 18.41a | 16.36a |
| Sorbitol | 4.45a | 11.57b | Histidine | 87.88a | 84.34a |
| Raffinose | 4.22a | 18.78b | Alanine | 222.87b | 75.91a |
| Aspartic acid | 46.18a | 87.97b | Tyrosine | 701.21b | 19.99a |
| Serine | 22.49a | 96.38b | Valine | 23.21a | 18.87a |
| Glycine | 311.72a | 351.64b | Methionine | 16.23a | 12.24a |
| Arginine | 22.73a | 42.50b | Lysine | 110.50b | 82.76a |
| Threonine | 3058.80a | 5197.32b |  |  |  |
| Proline | 1814.40a | 3638.44b |  |  |  |
| Cysteine | 40.75a | 124.73b |  |  |  |
| Isoleucine | 17.64a | 149.70b |  |  |  |
| Leucine | 12.79a | 47.76b |  |  |  |
| Phenylalanine | 21.47a | 55.12b |  |  |  |

Note: All values are mean of three (n = 6×3) experiments

Letters (a, b) in the sub-table for each item indicate significant difference at 4⁰C and 28⁰C incubation temperature
